# A clinical complexity of olanzapine use: Peripheral edema

**DOI:** 10.1111/bdi.13477

**Published:** 2024-07-30

**Authors:** Vincent Zhang, Mary‐Anne Hennen, Hector Lisboa, Najeeb Hussain

**Affiliations:** ^1^ Department of Psychiatry New Jersey Medical School Newark New Jersey USA

**Key Message**

Olanzapine is a potent atypical antipsychotic, which may lead to peripheral edema. Such edema can affect patients regardless of age, dose, or psychiatric conditions. Due to its rare presentation, this can be difficult to recognize and manage, especially in settings where assessing the patient head‐to‐toe is less easily done.

## 1 CASE PRESENTATION

The patient is a 19‐year old boy with a past medical history of bipolar I disorder, benzodiazepine use, and marijuana use disorder who presents after threatening his mother with a knife following an argument with both his parents. At home, he was not taking any prescribed medications. Upon admission to the inpatient psychiatry unit for mood dysregulation, he was started on paliperidone and divalproex sodium and clonazepam daily. Previous trials of risperidone and divalproex sodium were unsuccessful in the past without any side effects. After being admitted, he denied any audiovisual hallucinations/suicidal or homicidal ideation but was superficially cooperative, and his behavior was still erratic, labile, and violent. Indeed, the following day, he punched another patient in the face unprovoked, necessitating the addition of PRN medications (ziprasidone, diphenhydramine, and lorazepam) and being brought to the quiet room. Olanzapine was added and increased due to continued violence (banging his head against a wall and attacking a staff member). In spite of these various medications, the patient expressed poor insight into his condition, in addition to his need for medications and further treatments.

Just over a month following the arrival to the unit, the patient developed bilateral upper and lower extremity swelling along with redness. Per the patient, he had noticed such swelling days after he was first given medication in the hospital—however, he did not mention this, nor was it noticed or documented. This swelling manifested as bilateral pitting edema primarily in the lower extremities up to the knee, alongside additional redness of the fingers. The patient denied any associated shortness of breath, rash, pruritus, wheezing, history of allergies/urticaria/asthma and any other medical conditions (cardiac, renal, etc.). Medical workup was initiated, and laboratories were unremarkable besides a mild normocytic anemia. Cardiac and pulmonary physical examinations (and echocardiogram) revealed no pertinent positives, and the patient saturated well on room air throughout.

The development of edema can be attributed to the introduction of olanzapine, as seen by its onset a few days after initiation and disappearance following discontinuing the medication. All additional potential causes of the edema including cardiovascular, pulmonary, and allergic were ruled out as shown above. Furthermore, some of the other medications (divalproex sodium, paliperidone) the patient was taking at the time rarely cause edema, and the patient has no past history of such symptoms despite taking them before. Olanzapine was thereafter discontinued and replaced with chlorpromazine, and the patient's legs were elevated, and a trial of compression socks was started. And a week later, the patient's edema had all but subsided and his behavior and mentation were much improved without any aggression or violence. He was thus discharged following a verbal commitment to returning to outpatient treatment, taking his prescribed medications, and controlling his emotions.

## 2 DISCUSSION

Olanzapine is a second‐generation atypical antipsychotic initially approved for the management of psychotic disorders such as schizophrenia, but later approved for the management of mood disorders such as bipolar disorder and treatment‐resistant depression. Pharmacologically, it acts primarily at the serotonin (5H2) and dopamine (D2) receptors in the brain, blocking both.^1^ Through doing so, in the mesolimbic dopaminergic pathway, olanzapine can help reduce the positive symptoms of psychosis such as hallucinations, delusions, and thought disorganization. However, common side effects of olanzapine include undesired weight gain (and subsequent risk of metabolic effects), sedation (likely secondary to additional antihistamine activity), constipation, and dizziness.^2^

Premarket trials revealed that peripheral edema, though rare, occurred in approximately 3% of patients taking olanzapine, as opposed to 1% of patients taking a placebo.^3^ There have been multiple reports delineating such in adults who are receiving olanzapine for the treatment of their psychosis—it has never been described before in an adolescent and only once before in the treatment of bipolar disorder. Prior research has postulated pathways to explain such phenomena, including the vasodilation of blood vessels and subsequent decrease in vascular resistance, due to the additional antagonism of alpha‐1 receptors. Regardless of mechanism or specific drug, the development of edema with atypical antipsychotic usage is always managed in a similar manner: discontinuation of the offending agent and switching a well‐tolerated, preferably typical antipsychotic (to reduce the likelihood of edema occurring again in the future).^3^

It has previously been established that edema following olanzapine use can develop within days (as seen with our patient) or even months. Edema can happen at any dose, yet preliminary findings suggest that the risk is higher as the dose increases.^4^ Although self‐limiting and fairly benign, the edema could theoretically have gone on indefinitely if not accidentally noticed. In other words, from a public health perspective, our case illustrated a *near miss*—it was fortunate that the patient was in the hospital, wearing a short‐sleeve gown (which readily exposed the redness and swelling of his hands and forearms).

Accordingly, an emphasis on patient education and rapport should be highlighted. The patient should have been made aware of the potential for side effects like this, and if he felt more comfortable with the treatment team, he may have brought up these unwelcome changes on his own. Further complicating matters, it has been well‐established previously that adolescent patients tend to be less open to discussing physical changes in their body with healthcare professionals.^5^ In short, a culture of mutual trust needs to be facilitated so that patients are not only educated about potential complications from the medications they take but also comfortable enough to bring attention to them. If not, this will likely lead to healthcare mistrust and medication noncompliance. Our case also shows the importance of a visual or physical exam, no matter how brief. Patients may be unable to recognize side effects of certain psychiatric medications including edema, weight gain, and rashes. The onus is on the provider to parse them out. Particularly, in an outpatient setting or telepsychiatry though, this can be more difficult due to clothing, less eyes on the patient etc. Here, providers should be aware of these limitations and err toward caution.

This presentation is the first to illustrate a rare side effect of olanzapine in an adolescent and the second while treating bipolar disorder. Additional research needs to be done to assess the risk in children and adolescents, given that the possibility in adults is already well‐established. Although it is a reversible finding, provider awareness and patient education are key. Patients may not necessarily be able to recognize such symptoms or in certain populations such as adolescents, not as comfortable to discuss them. And in an outpatient or telepsychiatry setting, we believe that doing so as a provider can be even more difficult, necessitating all the more attention.

## 3 LEARNING POINTS

- Olanzapine can lead to peripheral edema through the vasodilation of blood vessels secondary to antagonism of alpha‐1 receptors.
- Patients must be educated given that peripheral edema can occur regardless of dose or purpose of the atypical antipsychotic and can affect patients of all ages.
- Providers should regularly conduct visual head‐to‐toe assessments or better yet a physical examination, given the easily reversible nature of such symptoms.

## CONFLICT OF INTEREST STATEMENT

All authors declare that they have no conflicts of interest.

## ETHICS STATEMENT

Informed consent was obtained from the patient, and identifiers were anonymized. Ethics approval from our ethics committee was waived due to the manuscript being a case report.

## Data Availability

The data that support the findings of this study are available on request from the corresponding author. The data are not publicly available due to privacy or ethical restrictions.
